# Supplementary material for: Integrated Hfq-interacting RNAome and transcriptomic analysis reveals complex regulatory networks of nitrogen fixation in root-associated Pseudomonas stutzeri A1501
Source: mSphere. 2024 May 15;9(6):e00762-23. doi: 10.1128/msphere.00762-23 (PMC11332353; doi:10.1128/msphere.00762-23)
Supplement: Supplemental material — Fig. S1 to S5; Table S3. [file msphere.00762-23-s0001.pdf]

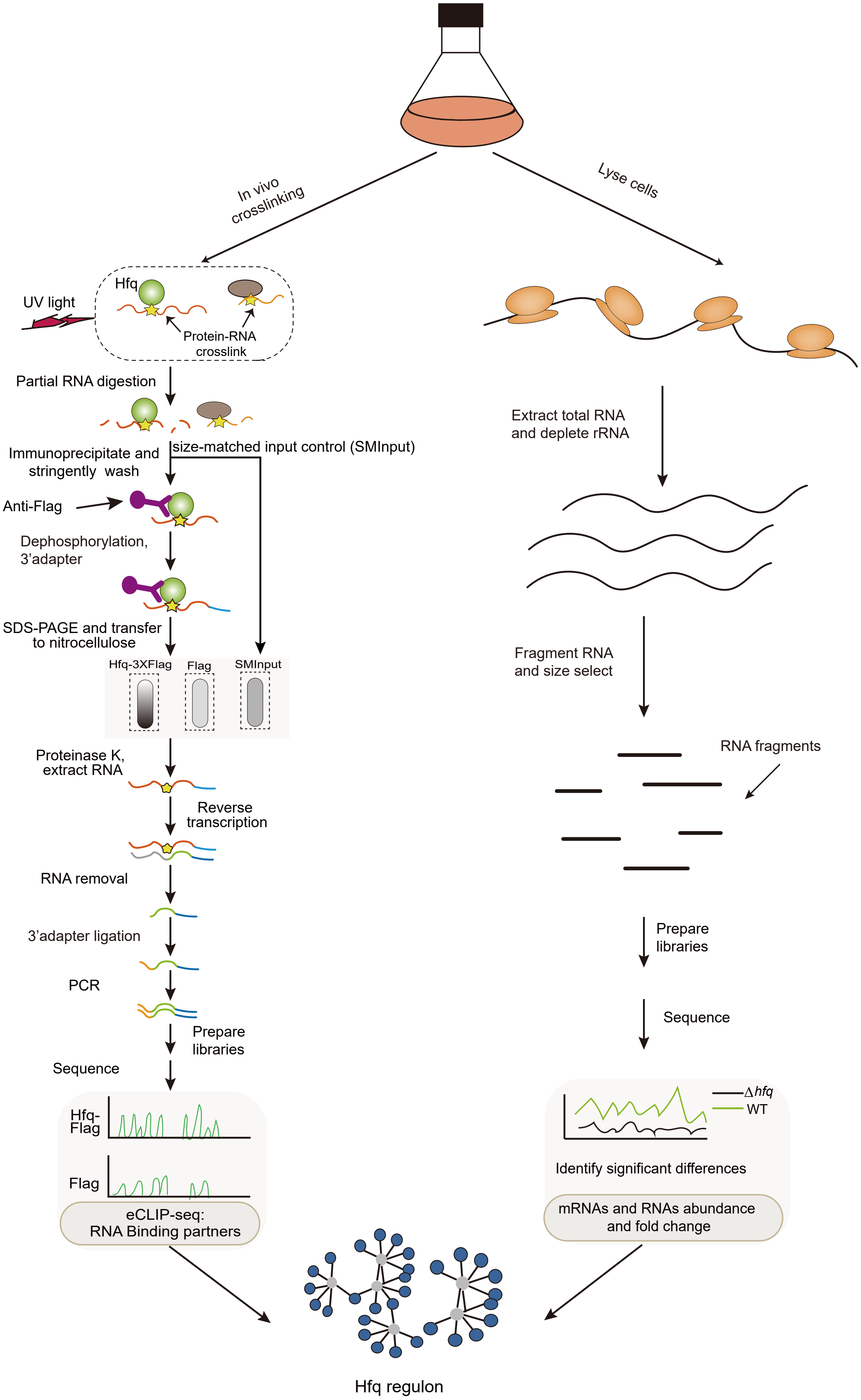

In vivo crosslinking
Lyse cells
UV light
Hfq
Protein-RNA crosslink
Partial RNA digestion
Immunoprecipitate and stringently wash
size-matched input control (SMInput)
Anti-Flag
Dephosphorylation, 3'adapter
SDS-PAGE and transfer to nitrocellulose
Hfq-3XFlag
Flag
SMInput
Proteinase K, extract RNA
Reverse transcription
RNA removal
3'adapter ligation
PCR
Prepare libraries
Sequence
Hfq-Flag
Flag
eCLIP-seq: RNA Binding partners
Extract total RNA and deplete rRNA
Fragment RNA and size select
RNA fragments
Prepare libraries
Sequence
Δhfq
WT
Identify significant differences
mRNAs and RNAs abundance and fold change
Hfq regulon

Fig. S1: Overview of integrated transcriptomic approaches.

Two transcriptomic methods were used to investigate the role of Hfq in gene expression at 6 h after inoculation under nitrogen fixation: eCLIP-seq (left) and RNA-seq (right). For eCLIP-seq analysis, the cells were treated with UV light to induce crosslinks and subjected to RNase I digestion, immunoprecipitation of Hfq-RNA complexes with Flag antibody, and stringent washes. After the dephosphorylation of RNA fragments, a barcoded RNA adapter was ligated to the 3’ end. The protein-RNA complexes were separated by SDS-PAGE and then transferred to a nitrocellulose membrane. A 75-kDa region greater than that of Hfq was excised, treated with proteinase K to isolate RNA, and further purified to generate sequence libraries. For RNA-seq, cells were collected under the same conditions as mentioned above. RNA was isolated and sequenced.

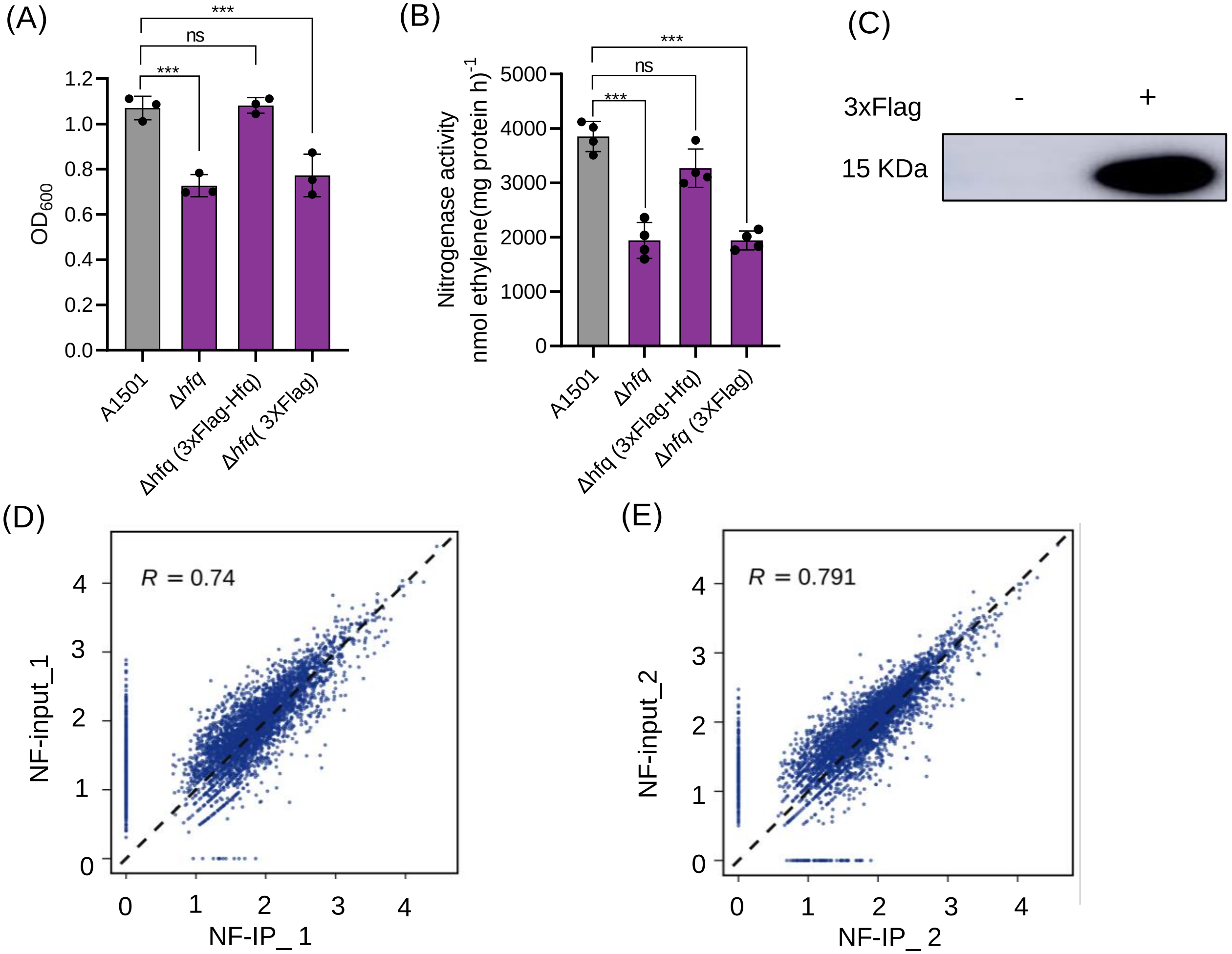

Fig. S2 3xFlag insertion downstream of *hfq* did not impair bacterial physiology. (A). Growth rate of wild-type, Δ*hfq*, Δ*hfq* (3xFlag-Hfq), and Δ*hfq* (3xFlag) strains. (B). The nitrogenase activity of wild-type, Δ*hfq*, Δ*hfq* (3xFlag-Hfq), and Δ*hfq* (3xFlag) strains. (C). Western blot of Hfq-RNA complex separated by SDS-PAGE by using anti-FLAG monoclonal antibody. (D, E) Correlation analysis based on RNA classes from total RNA sequencing. Coefficients of determination R and principal components between Input and IP were calculated from RNA sequencing.

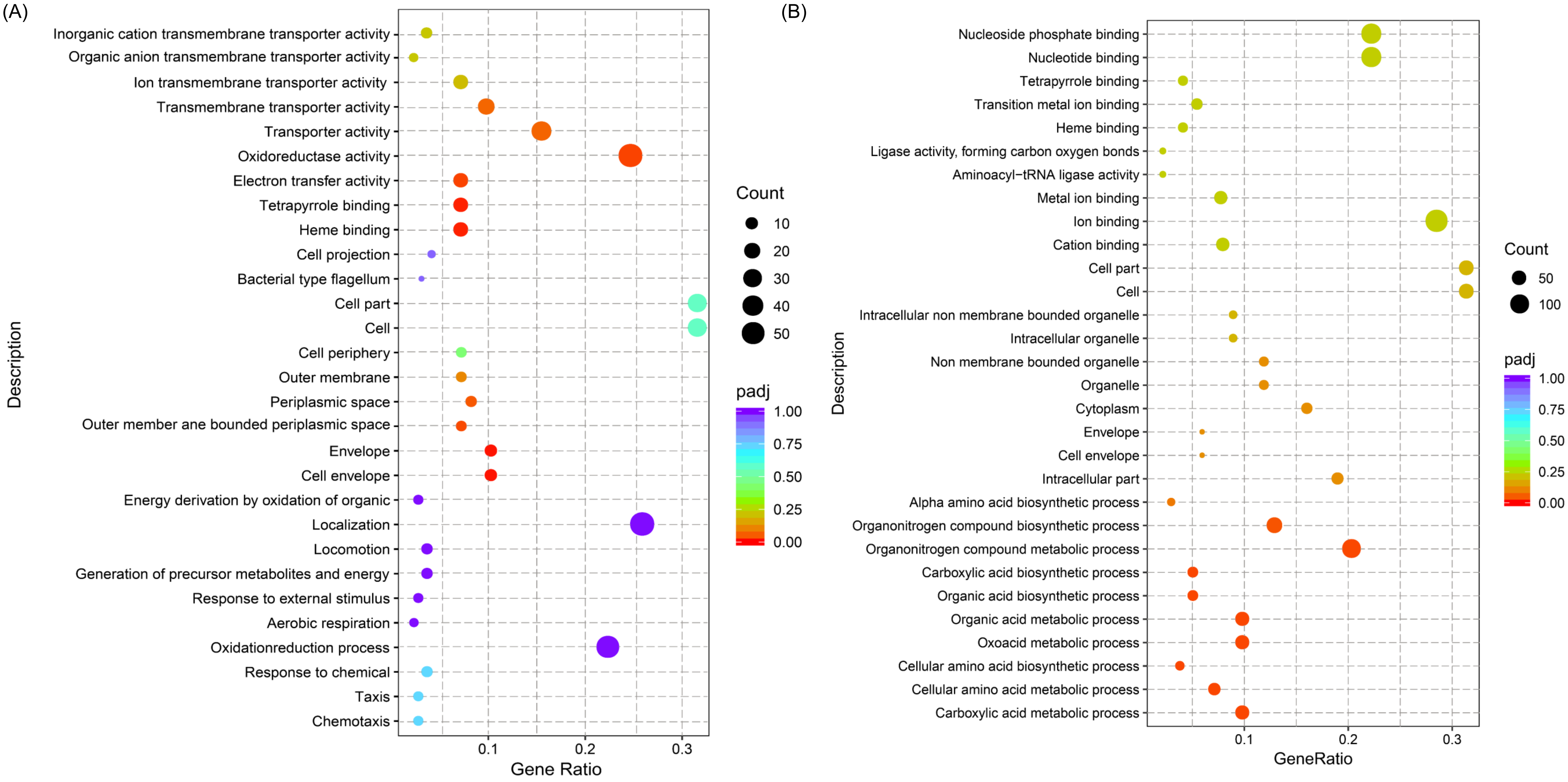

Fig. S3 Gene ontology (Go) enrichment analysis of genes in Hfq eCLIP-seq peaks (A) and differentially expression genes between the WT and *hfq* mutant strain in RNA-seq (B).

(A)

(B)

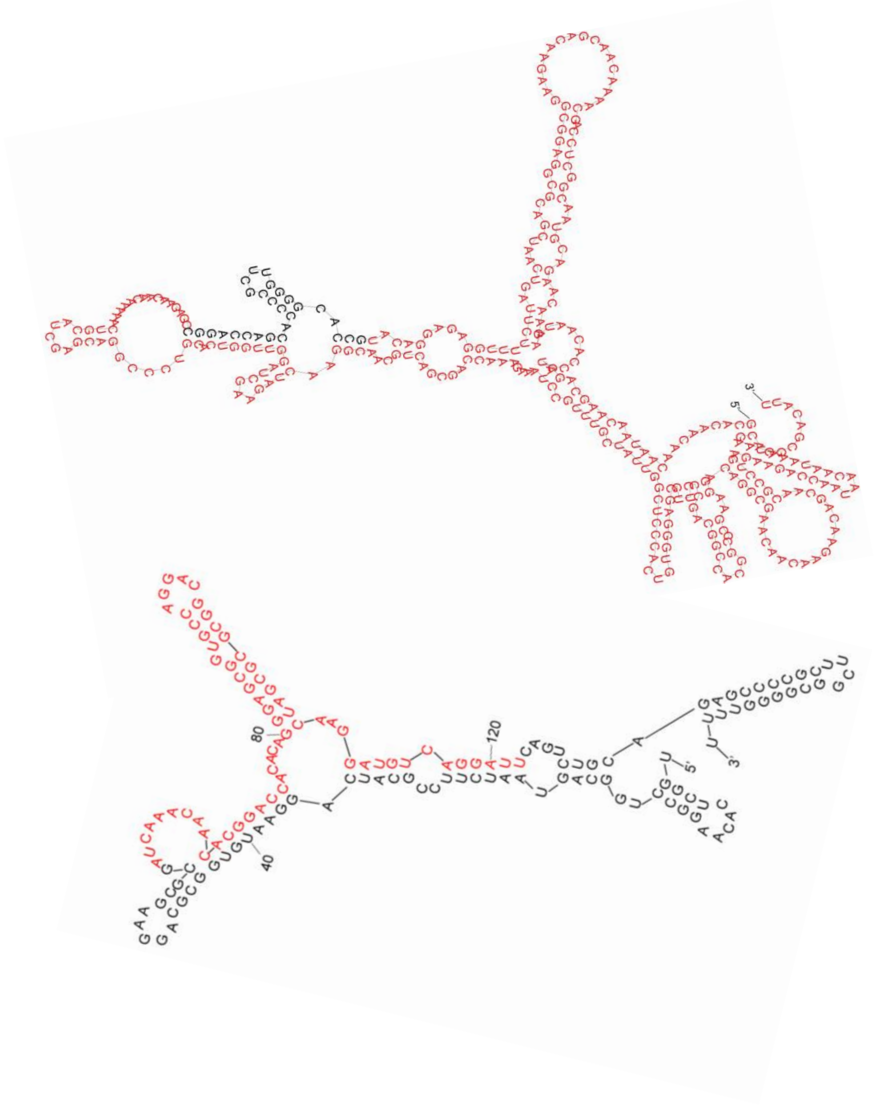

Fig. S4 A.B Secondary structures of Hfq binging sites at the CrcZ (A) and RsmY (B) was predicted using RNA fold. In the RNAs structures, the red letters indicate Hfq cross-linking sites.

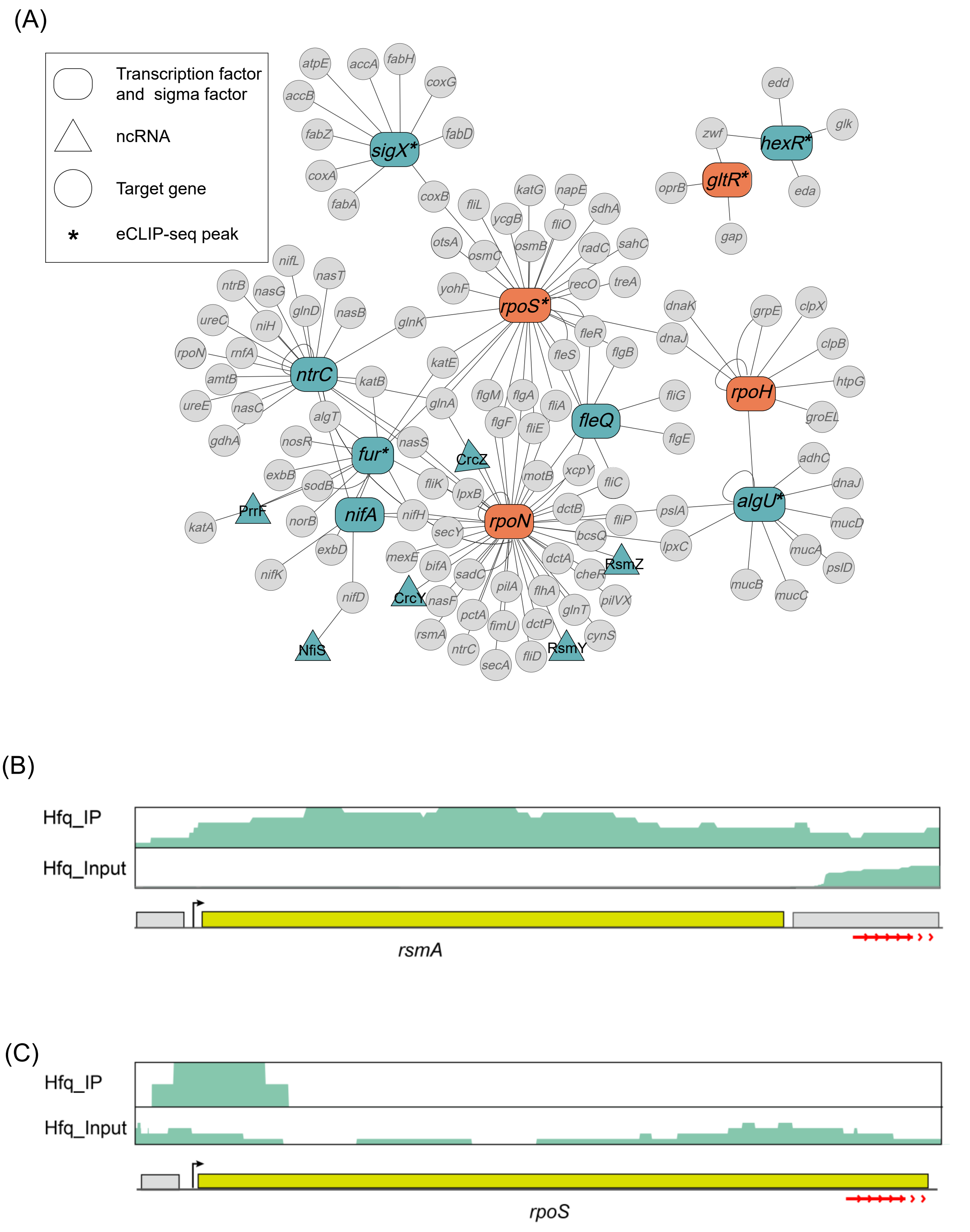

Fig. S5 A. Transcriptional regulators, sigma factors, and ncRNAs that are differentially expressed in the hfq mutant and their target genes are shown. Regulators represented as squares are TFs and sigma factors, and triangles are ncRNAs. The asterisks indicate that a Hfq eCLIP-seq peak is associated with the gene.Rads coverage at the *rsmA* (B) ans *rpoS* (C) loci in libraries from Hfq eCLIP-seq data in Hfq_IP and Hfq_SMInput samples.

Table S3 Different expression of ncRNAs in Δ*hfq* compare with WT

gene_id	name	start	end	length	log2Fold Change	p-value
ncRNA_001	NfiS	32731	33132	401	-3.519	-0.01
ncRNA_002	ncRNA_002	80908	81171	263	0.85	-0.01
ncRNA_003	NcRNA_004	251790	252121	331	-2.79	-2.19E-10
ncRNA_004	ncRNA	440206	440464	259	-1.61	0.00013009
ncRNA_005	NcRNA_007	601735	602061	326	1.67	-0.03
ncRNA_006	ncRNA	621059	621110	52	-3.28	1.06E-12
ncRNA_007	NcRNA_008	641281	641361	81	-3.28	3.43E-09
ncRNA_008	NcRNA_008	641382	641633	252	-2.39	0.00118483
ncRNA_009	NcRNA_008	641523	641705	182	-2.66	-9.50E-09
ncRNA_010	ncRNA	652213	652454	242	-3.07	9.16E-12
ncRNA_011	ncRNA	655065	655345	281	-1.98	4.19E-06
ncRNA_012	ncRNA	672611	672750	140	-3.19	3.16E-12
ncRNA_013	ncRNA	673064	673314	251	-3.51	2.47E-14
ncRNA_014	ncRNA	675783	676040	258	-2.32	1.61E-07
ncRNA_015	ncRNA	718394	718599	206	-3.47	4.48E-14
ncRNA_016	NcRNA_009	725599	725922	323	-2.69	-7.49E-10
ncRNA_017	NcRNA_009	725623	725892	270	-2.81	2.37E-10
ncRNA_018	ncRNA	725995	726192	198	-2.24	2.80E-07
ncRNA_019	NcRNA_010	733074	733340	266	-2.15	-6.24E-05
ncRNA_020	ncRNA	734418	734635	218	-2.60	4.01E-09
ncRNA_021	ncRNA	739475	739977	503	-4.20	7.19E-19
ncRNA_022	ncRNA	742706	742969	264	-14.43	9.03E-46
ncRNA_023	ncRNA	754185	754380	196	-4.16	3.62E-18
ncRNA_024	NcRNA_012	770571	770753	182	-3.25	-2.65E-10
ncRNA_025	ncRNA	800775	801064	290	-2.31	1.11E-07
ncRNA_026	ncRNA	876198	876317	120	-3.25	1.39E-12
ncRNA_027	ncRNA	898866	898989	124	-1.62	0.00038162
ncRNA_028	ncRNA	1056960	1057046	87	-2.13	0.02307085
ncRNA_029	ncRNA	1073573	1073746	174	-2.61	3.96E-09
ncRNA_030	ncRNA	1075036	1075108	73	-4.08	2.60E-17
ncRNA_031	ncRNA	1078713	1078805	93	-2.73	1.15E-09
ncRNA_032	ncRNA	1080421	1080632	212	-3.28	5.95E-13
ncRNA_033	Rnase P	1180839	1181198	359	-0.56	-0.17
ncRNA_034	ncRNA	1200438	1200582	145	-9.18	3.92E-10
ncRNA_035	NcRNA_019	1280032	1280143	111	-4.34	-5.53E-18
ncRNA_036	NcRNA_020	1321220	1321642	422	1.32	-0.055
ncRNA_037	ncRNA	1331623	1331905	283	-2.00	3.20E-06
ncRNA_038	NcRNA_021	1343148	1343354	206	5.02	-0.13
ncRNA_039	NcRNA_023	1414065	1414457	392	2.23	-0.191

ncRNA_040	ncRNA	1434826	1435001	176	-2.77	4.83E-10
ncRNA_041	ncRNA	1471339	1471432	94	-1.99	5.05E-06
ncRNA_042	ncRNA	1471433	1471714	282	-5.48	1.13E-27
ncRNA_043	ncRNA	1471648	1471697	50	-6.11	0.040964585
ncRNA_044	ncRNA	1471801	1471909	109	-2.25	1.33E-06
ncRNA_045	ncRNA	1478196	1478355	160	-2.45	2.48E-08
ncRNA_046	ncRNA	1485723	1486237	515	-1.77	3.07E-05
ncRNA_047	ncRNA	1495317	1495466	150	-2.96	5.04E-11
ncRNA_048	ncRNA	1498177	1498474	298	-3.86	8.58E-17
ncRNA_049	ncRNA	1506171	1506264	94	-3.80	1.08E-15
ncRNA_050	ncRNA	1506794	1507039	246	-2.76	4.24E-10
ncRNA_051	ncRNA	1564576	1564940	365	-1.98	3.49E-06
ncRNA_052	ncRNA	1591539	1591747	209	-2.08	1.56E-06
ncRNA_053	ncRNA	1591949	1592154	206	-5.10	7.03E-24
ncRNA_054	NcRNA_015	1609646	1609877	231	-2.30	-6.35E-07
ncRNA_055	ncRNA	1632342	1632423	82	-3.69	9.65E-16
ncRNA_056	ncRNA	1692249	1692369	121	-3.81	2.21E-15
ncRNA_057	ncRNA	1698365	1698583	219	-1.55	0.000240007
ncRNA_058	ncRNA	1751497	1751726	230	-2.78	4.21E-10
ncRNA_059	ncRNA	1752722	1752933	212	-1.98	8.48E-06
ncRNA_060	ncRNA	1903877	1904209	333	-4.67	4.07E-22
ncRNA_061	ncRNA	1904187	1904568	382	-1.72	4.74E-05
ncRNA_062	ncRNA	1961727	1962077	351	-2.28	1.14E-07
ncRNA_063	ncRNA	2010390	2010779	390	-2.03	1.85E-06
ncRNA_064	ncRNA	2011964	2012361	398	-2.20	3.46E-07
ncRNA_065	ncRNA	2016105	2016309	205	-2.65	2.62E-09
ncRNA_066	ncRNA	2019327	2019675	349	-2.19	3.80E-07
ncRNA_067	ncRNA	2019376	2019450	75	-2.95	1.50E-10
ncRNA_068	CrcY	2072694	2073137	443	-5.96	-8.80E-32
ncRNA_069	CrcY	2072813	2073130	318	-5.82	1.46E-29
ncRNA_070	ncRNA	2214870	2215138	269	-3.89	3.90E-17
ncRNA_071	ncRNA	2241627	2241890	264	-3.61	2.92E-15
ncRNA_072	NcRNA_032	2251285	2251356	71	-1.65	-0.3
ncRNA_073	ncRNA	2375866	2376026	161	-1.80	2.98E-05
ncRNA_074	ncRNA	2510011	2510223	213	-2.52	1.01E-08
ncRNA_075	ncRNA	2595806	2595960	155	-3.30	7.57E-13
ncRNA_076	ncRNA	2596033	2596380	348	-3.53	1.00E-14
ncRNA_077	ncRNA	2718407	2718546	140	-3.35	3.24E-13
ncRNA_078	ncRNA	2728585	2728726	142	-4.24	1.47E-19
ncRNA_079	ncRNA	2728676	2729125	450	-2.80	1.80E-10
ncRNA_080	ncRNA	3024659	3025000	342	-2.94	3.95E-11
ncRNA_081	ncRNA	3039468	3039552	85	-2.69	1.17E-09

ncRNA_082	NcRNA_039	3220711	3221151	440	5.02	-0.1
ncRNA_083	ncRNA	3444783	3445178	396	-3.61	2.86E-15
ncRNA_084	ncRNA	3446774	3447058	285	-3.33	1.73E-13
ncRNA_085	ncRNA	3447569	3447681	113	-4.06	3.19E-17
ncRNA_086	ncRNA	3447867	3448124	258	-3.30	3.00E-13
ncRNA_087	CrcZ	3565453	3565781	329	-10.50	-6.09E-64
ncRNA_088	ncRNA	3618156	3618528	373	-2.39	3.22E-08
ncRNA_089	ncRNA	3623586	3623801	216	-3.04	2.59E-11
ncRNA_090	ncRNA	3626504	3627233	730	-2.25	1.74E-07
ncRNA_091	ncRNA	3634973	3635470	498	-3.22	6.79E-13
ncRNA_092	ncRNA	3649550	3649709	160	-3.05	1.48E-11
ncRNA_093	ncRNA	3658891	3659160	270	-2.64	3.15E-09
ncRNA_094	ncRNA	3667602	3668012	411	-2.22	2.51E-07
ncRNA_095	ncRNA	3670773	3671272	500	-2.14	5.91E-07
ncRNA_096	ncRNA	3670776	3671168	393	-1.77	2.88E-05
ncRNA_097	ncRNA	3691561	3692010	450	-1.86	1.31E-05
ncRNA_098	ncRNA	3703558	3703916	359	-2.36	5.45E-08
ncRNA_099	ncRNA	3721339	3721564	226	-3.56	1.06E-14
ncRNA_100	ncRNA	3736703	3737142	440	-2.09	1.11E-06
ncRNA_101	ncRNA	3738780	3738845	66	-3.00	1.64E-11
ncRNA_102	ncRNA	3739445	3739851	407	-2.09	1.20E-06
ncRNA_103	ncRNA	3812560	3812693	134	-4.90	5.07E-20
ncRNA_104	ncRNA	3817134	3817224	91	-3.63	5.34E-15
ncRNA_105	ncRNA	3963695	3964067	373	-10.36	2.56E-53
ncRNA_106	ncRNA	3963889	3963980	92	-16.01	1.26E-57
ncRNA_107	RsmY	4177619	4177817	199	-7.94	3.25E-43
ncRNA_108	ncRNA	4405838	4405994	157	-1.66	0.000102948
ncRNA_109	NcRNA_056	4429655	4429833	178	-2.31	-1.65E-06
ncRNA_110	ncRNA	4500263	4500509	247	-4.06	9.40E-18

+, With putative binding site; -, Without putative binding site

Table S4 Strains and plasmids used in this study

Strain/plasmid	Genotypes and relevant features	Reference
**Strain**		
A1501	Wild type, Chinese culture collection: CGMCC 0351	
Δ*hfq*	A1501 devoid of *PST3668*; Hyg and Cm	This study
Δ*hfq* (pL*hfq*)	Δ*hfq* containing pL*hfq*, Tc, Cm and Hyg	This study
Δ*hfq* (3xFlag-Hfq)	Δ*hfq* containing pL*hfq*-3xFlag, Tc, Cm and Hyg	This study
Δ*hfq* (3xFlag)	Δ*hfq* containing pL3xFlag, Tc, Cm and Hyg	This study
**Plasmids**		
pRK2013	Helper plasmid, ColE1 replicon, Tra; Km	Lab collection
pK18*mobsacB*	Suicide plasmid for gene deletion mutation, Km	Lab collection
pLAFR3	Broad-host-range plasmid, Tc	Lab collection
pUC18-mini-Tn7-Gm-lacZ	Mini-Tn7 vector, Amp, Gm	Choi et al. 2005
pUX-BF13	Helper plasmid for transposition of mini-Tn7 element, Amp	Liu et al. (2014)
pXY2	Mini-Tn7 vector for a lacZ translational fusion, Gm	Liu et al. (2014)
Mini pCrcZ	Mini-Tn7 vector containing the P_{crcZ} promoter, Gm	This study
pXY2-P*zwf*	pXY2 containing the P*zwf-lacZ* transitions fusion, Gm	This study
pXY2-PΔCA *zwf*	pXY2 containing the PΔCA *zwf -lacZ* transitions fusion, Gm	This study
pXY2-P*gcd*	pXY2 containing the P*gcd-lacZ* transitions fusion, Gm	This study
pXY2-PΔCA *gcd*	pXY2 containing the PΔCA *gcd -lacZ* transitions fusion, Gm	This study
pXY2-P*oprB*	pXY2 containing the P*oprB-lacZ* transitions fusion, Gm	This study
pXY2-PΔCA*oprB*	pXY2 containing the PΔCA *oprB -lacZ* transitions fusion, Gm	This study
pXY2-P*gtsA*	pXY2 containing the P*gtsA-lacZ* transitions fusion, Gm	This study
pXY2-PΔCA*gtsA*	pXY2 containing the PΔCA *gtsA-lacZ* transitions fusion, Gm	This study
pXY2-P*citE*	pXY2 containing the P*citE-lacZ* transitions fusion, Gm	This study
pXY2-P*glnA*	pXY2 containing the P*glnA-lacZ* transitions fusion, Gm	This study
pXY2-P*nifA*	pXY2 containing the P*nifA-lacZ* transitions fusion, Gm	This study
pXY2-P*nifH*	pXY2 containing the P*nifH-lacZ* transitions fusion, Gm	This study
pXY2-P*cheZ*	pXY2 containing the P*cheZ-lacZ* transitions fusion, Gm	This study
pXY2-P*cheR*	pXY2 containing the P*cheR-lacZ* transitions fusion, Gm	This study
pXY2-P*fliG*	pXY2 containing the P*fliG-lacZ* transitions fusion, Gm	This study
pXY2-P*rpoS*	pXY2 containing the P*rpoS-lacZ* transitions fusion, Gm	This study
pXY2-P*algU*	pXY2 containing the P*algU-lacZ* transitions fusion, Gm	This study
pXY2-P*rpoN*	pXY2 containing the P*rpoN-lacZ* transitions fusion, Gm	This study
pXY2-P*fleQ*	pXY2 containing the P*fleQ-lacZ* transitions fusion, Gm	This study
pXY2-P*fur*	pXY2 containing the P*fur-lacZ* transitions fusion, Gm	This study
pXY2-P*ntrC*	pXY2 containing the P*ntrC-lacZ* transitions fusion, Gm	This study
pXY2-P*sigX*	pXY2 containing the P*sigX-lacZ* transitions fusion, Gm	This study
pXY2-P*gltR*	pXY2 containing the P*gltR-lacZ* transitions fusion, Gm	This study
pXY2-P*hexR*	pXY2 containing the P*hexR-lacZ* transitions fusion, Gm	This study
pXY2-P*rpoH*	pXY2 containing the P*rpoH-lacZ* transitions fusion, Gm	This study
pL*hfq*	pLAFR3 carrying A1501 *hfq* encoding sequence under control of its authentic promoter	This study
pL*hfq*-3xFlag	pLAFR3 carrying A1501 *hfq* encoding sequence under control of its authentic promoter	This study
pL3xFlag	pLAFR3 carrying 3xFlag	This study

Table S5 Oligonucleotides used in this study

Name	Sequences (5'-3')
hfq-3xFlag F	ACAGCTATGACCATGATTACGAATTCAAATGGTGGAGCGCGGCAT
hfq-3xFlag R	CGACGGCCAGTGCCAAGCTTTCACTTGTCATCGTCATCCTTGTAGTCGATGT CATGATCTTTATAATCACCGTCATGGTCTTTGTAGTCGTCGTTACCCGACTC
3xFlag F	GCTATGACCATGATTACGAATTCGACTACAAAGACCATG
3xFlag R	CGACGGCCAGTGCCAAGCTTCTTGTCATCGTC
PcrcZ-lacZ-F	gatcatgcatgagctcaCCGGGTAATTTGTTACCG
PcrcZ -lacZ-R	gaggtaccgggcccaGTTGTTGTGCCATTAGGTAAG
gcd-lacZ-F	attcgatcatgcatgagctcactagtCCGCCTCATCACGTCTGG
gcd-lacZ-R	tcacgacgttgtaaaacgacaagcttCAATCCGCTGATGACTACTGCC
ΔCA*gcd*-lacZ-R	cgacgttgtaaaacgacaCAATCCGCTGATGACTACTGCCACGGCGCCAACTCCTCGCTCgctactga CGAC
zwf-lacZ-F	attcgatcatgcatgagctcactagtCTCTACCGCCTGGCTGACA
zwf-lacZ-R	tcccagtcacgacgttgtaaaacgacaGGGTTCGACAGATAATGGC
ΔCA*zwf*-lacZ-R	tcccagtcacgacgttgtaaaacgacaGGGTTCGACAGATAATGGCGTCAtgatgtgctACTGACTAC
oprB-lacZ F	attcgatcatgcatgagctcactagtGACCGGGAGCTCATTCTGG
oprB-lacZ R	tcacgacgttgtaaaacgacaagcttCAGGGCTGTCAGTGTTTTTTTCATATCC
ΔCA*oprB*-lacZ-R	tcacgacgttgtaaaacgacaagcttCAGGGCTGTCAGTGatatattCATATCC
gtsA-lacZ F	attcgatcatgcatgagctcactagtACCAGACCCGCGTGAC
gtsA-lacZ R	tcacgacgttgtaaaacgacaagcttGTCTATGCCCGGAAAAATGGACAC
ΔCA *gtsA*-lacZ-R	tcacgacgttgtaaaacgacaagcttGTCTATGCCCGGaaaaatgGACAC
citE-lacZ F	attcgatcatgcatgagctcactagtAGTGAAGTCGACAGCCCGTC
citE-lacZ R	tcacgacgttgtaaaacgacaagcttATGAGTTCATGGGCTGGCTCC
glnA-lacZ F	attcgatcatgcatgagctcactagtCTTGCGCACCGGCGGGCTCTTG
glnA -lacZ R	tcacgacgttgtaaaacgacaagcttTCTTTGATCAGTTGAAGCGACTTCGACATG
nifH-lacZ F	attcgatcatgcatgagctcactagtCCAGTGGATTTACCGATAGCCG
nifH -lacZ R	tcacgacgttgtaaaacgacaagcttCCGTAAATAGCGCATTGACGCATTGCC
nifA-lacZ F	attcgatcatgcatgagctcactagtACTGGCAGCCGGCGCTGCGCCTAC
nifA-lacZ R	tcacgacgttgtaaaacgacaagcttCTGGGGCGTTCGGCGAATGTGGCGTTCATGC
rpoS-lacZ F	attcgatcatgcatgagctcactagtAAGGAATTGATATCGCTGG
rpoS-lacZ R	tcacgacgttgtaaaacgacaagcttAACTCCAGCGCTTGGTCTTTAAGTGCC
algU-lacZ F	attcgatcatgcatgagctcactagtTAGGCAGTTATGAATGCAAACCAG
algU-lacZ R	tcacgacgttgtaaaacgacaagcttAGTTGCTGGTCCTGCTCCTGAGTCAGCATG
rpoN-lacZ F	attcgatcatgcatgagctcactagtCGGCTCGGCAAATCGGCTCTAGGCAA
rpoN -lacZ R	tcacgacgttgtaaaacgacaagcttTCGCCGCTGTGCGAAGGCTCTTCATA
fleQ-lacZ F	attcgatcatgcatgagctcactagtGCGAACTGCGTCAGCTCAATC
fleQ-lacZ R	tcacgacgttgtaaaacgacaagcttAGAGGCTGAAAGAAAGCCTGCGCTCGCATC
fur-lacZ F	attcgatcatgcatgagctcactagtGCCAGCTGGTCGTTGGCATTG
fur-lacZ R	tcacgacgttgtaaaacgacaagcttGCTTTACGTAGTTCGCTATTTTCAACC
ntrC-lacZ F	attcgatcatgcatgagctcactagtACCCGAGCATCCCCGAGG
ntrC-lacZ R	tcacgacgttgtaaaacgacaagcttACGATCCAGACGTTTTCGCTTCGGCTCATGC
cheZ-lacZ F	attcgatcatgcatgagctcactagtACATGAAAATCCTCATCGTCGATG
cheZ-lacZ R	tcacgacgttgtaaaacgacaagcttTCTGCCAGGCTTTGGTCTGCTTGCGTCATAGTG

cheR-lacZ F	attcgatcatgcatgagctcactagtGATGGCCGACGACGGCAAGCG
cheR-lacZ R	tcacgacgttgtaaaacgacaagcttTTGTTCAGCCGGCTGGACACCAGATAC
fliG-lacZ F	attcgatcatgcatgagctcactagtAGCGTGATCAATACCGCCTTCGTCGC
fliG-lacZ R	tcacgacgttgtaaaacgacaagcttAGCTTGGCTGGAACTCGATTG
sigX-lacZ F	attcgatcatgcatgagctcactagtGGGGCTGGTGATCATCATCAG
sigX -lacZ R	tcacgacgttgtaaaacgacaagcttGTTTTAGTCAAGACGCTTCGAGCCGGCAATC
gltR-lacZ F	attcgatcatgcatgagctcactagtCGGGTTTGCTGACGCTCT
gltR-lacZ R	tcacgacgttgtaaaacgacaagcttGATGTTCTTACCTGCTTGGCTCAC
hexR-lacZ F	attcgatcatgcatgagctcactagtTCGCCGCCGTCGCGGGCGAGGCC
hexR-lacZ R	tcacgacgttgtaaaacgacaagcttCCTTGCGCTCGGCCTTGTTGAG
rpoH-lacZ F	attcgatcatgcatgagctcactagtGTCAAGCTGGTCGGCGGTACCGAC
rpoH lacZ R	tcacgacgttgtaaaacgacaagcttGCTTGAACAGGTTGCAGAGTAGTTGTCATGC
RT16S F	CCTACGGGAGGCAGCAG
RT16S R	ATTACCGCGGCTGCTGG
RT *crcZ* F	GGACATGCCACGGGGTTC
RT *crcZ* R	CGGATTTCTTTGGTCGCTGA
RT *rsmA F*	CCGGGTAGGAGAAACCCTGA
RT *rsmA R*	ATCCGCTGGTAAATCTCCTCG
RT *rpoS F*	GATCGAAAGCAACCTGCGAC
RT *rpoS R*	GGTCGCGTAGGTAGAGAAGC
RT *algU F*	ATGCCGAGTTCTACGAAGGC
RT *algU R*	CCATCAAATTCGCGCAGTGT
RT *rpoN F*	CTGCTCAGTCGCAACGAAAC
RT *rpoN R*	ATCGTCGACTCATGCATCCC
RT *rpoH F*	GGTCTGATGAAGGCGGTCAA
RT *rpoH R*	CGCAGGATGAACTCGTGGAT
RT *ntrC F*	TGATCAATGGCGAATCGGGT
RT *ntrC R*	CCGAACAGCTCGGATTCCAT
RT *fleQ F*	CCATCAGGTTCAACTCGGCT
RT *fleQ R*	AGACTGGTTGCGTACTGCTC
RT *rsmYF*	GCGATCAAACAACACGGACC
RT *rsmYR*	GGGCTCTGCAGACTGAATCC
RT *sadC F*	TGGCGAGTGGTTTCTACGAG
RT *sadC R*	ATAGAGCACGAGCAGTGAGC
RT *prrf F*	AGCCGATAGCTGAAGGCTCA
RT *prrf R*	CAGAGCCGGGTCAAAACCG
RT *cheZ F*	ATCGACCAGTATCTGCAGCG
RT *cheZ R*	GCTCAGTGATCAGTCGGGTC
RT *glnA F*	TCTCCAAGGACGGCAAGAAC
RT *glnA R*	ACGCTTGTAGGAGTTGGTCG
RT *nifA F*	CGCGAAGACCTCTACTACCG
RT *nifA R*	CAGCTTGAGTTTGCGACCCT
RT *nifH F*	GAGATGATGGCGATGTATGC
RT *nifH R*	GGTCGGTGTTGCGGCTGTTG

RT *fliG F*	TGTCCCACTTCGACCACAAG
RT *fliG R*	TCGATGGAGCTGTCCAGGTA
RT *cheR F*	GTTTCGCGATACCTACCCGT
RT *cheR R*	GTATACCGGTCTTCGGCTGG
RT *sigX F*	CATATTACGCGGGCCTACGA
RT *sigX R*	TTTCCCAAATAGCGGGCACA
RT *fur F*	AGGTCTCAAGGTAACGCTGC
RT *fur R*	GATAGACAGTCGCCAGACCG
RT *gltR F*	CGGCCGATCTGGTGATACTC
RT *gltR R*	GCATGATGATCGGCATCGAG
RT *hexR F*	TATGTCAGCCAGGCGGTAGA
RT *hexR R*	GCCGAAAAAGTGGATCTGGC

For the sequences listed for oligonucleotides, restriction sites are underlined, F. Forward; R. Reverse
